# Conduction Mechanism Analysis of Abrupt- and Gradual-Switching InGaZnO Memristors

**DOI:** 10.3390/mi13111870

**Published:** 2022-10-30

**Authors:** Woo Sik Choi, Min Suk Song, Hyungjin Kim, Dae Hwan Kim

**Affiliations:** 1 School of Electrical Engineering, Kookmin University, Seoul 02707, Korea; 2 Department of Electronic Engineering, Inha University, Incheon 22212, Korea

**Keywords:** memristor, InGaZnO, gradual and abrupt switching, conduction mechanism, oxygen vacancy, random telegraph noise

## Abstract

In this work, two types of InGaZnO (IGZO) memristors were fabricated to confirm the conduction mechanism and degradation characteristics of memristors with different electrode materials. The IGZO memristor exhibits abrupt switching characteristics with the Pd electrode owing to the formation and destruction of conductive filaments but shows gradual switching characteristics with the p-type Si electrode according to the amount of generated oxygen vacancy. The electrical characteristics and conduction mechanisms of the device are analyzed using an energy band diagram and experimentally verified with random telegraph noise characteristics confirming the trap effects on the device conduction.

## 1. Introduction

Recently, a neuromorphic system has been widely studied to supplement the computing efficiency of a von Neumann structure [1,2,3,4,5]. Unlike conventional computing architectures, it can process data efficiently thanks to a parallel structure, and quickly maximize energy efficiency and operation speed, increasing the feasibility of in-memory computing in which computations can be conducted inside memory devices. Nonvolatile memories, such as memristors, flash memories, and ferroelectric memories, are being studied as synapses to store weight values for vector-matrix multiplications [6,7,8,9,10,11,12,13,14,15]. Especially a memristor is one of the most potential candidates because of its fast switching speed, low operation voltage, and simple structure having two terminals, which leads to a cross-point array structure with superior advantages of a high-density parallel-connected structure [16,17,18,19,20,21,22,23,24,25].

Memristor devices can be divided into abrupt and gradual types based on their switching characteristics. An abrupt-switching memristor switches with the formation and collapse of a conductive filament (CF) constructed by oxygen vacancy (V_O_) and has binary conductance states [26,27,28,29]. Although abrupt-switching memristors have fast switching and good retention characteristics, they have limitations in terms of expressing the floating-point weight values of artificial neural networks and significant disadvantages in terms of endurance and device variation owing to the nonuniformly formed and ruptured CF [30,31]. In contrast, gradual-type devices are switched according to the amount of V_O_ generated near the interface between the electrode or switching layer, resulting in multiconductance states; they tend to have the disadvantages of poor retention characteristics and relatively slow switching speeds because of the interface-type conducting path [32,33,34,35,36,37,38,39,40].

In this study, we fabricated two types of devices with different electrode materials (Pd/InGaZnO(IGZO)/Pd and Pd/IGZO/SiO2/p-type Si), and their electrical characteristics are analyzed. IGZO is widely used as a channel material for display devices thanks to high mobility and uniformity and has a great advantage in cointegration with CMOS circuitry through a low-temperature process [41,42,43,44,45,46]. Depending on the bottom electrode, the abrupt and gradual switching characteristics are analyzed through an energy band diagram, and conduction mechanisms are verified by replotted current (*I*)–voltage (*V*) characteristics. In addition, the random telegraph noise (RTN) signals of both devices are analyzed to verify the presence or absence of the CF in each device.

## 2. Materials and Methods

**Figure 1:**
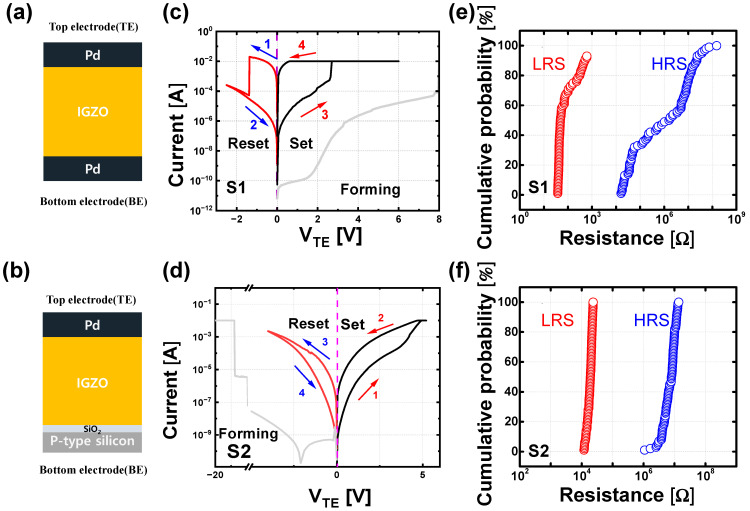
(**a**,**b**) Schematic view and (**c**,**d**) electrical switching *I*–*V* characteristics of S1 and S2 with a feature size of 10 μm. (**e**,**f**) Cumulative probability plot for both HRS and LRS of S1 and S2 measured at 0.1 V.

The two types of IGZO memristors are fabricated having an active area of 10 × 10 μm^2^ with the different electrodes, respectively, as shown in Figure 1a,b. Sample #1 (S1) has a Pd bottom electrode (BE), and sample #2 (S2) has p-type Si BE and an additional SiO_2_ layer. The bottom electrode (BE) was deposited using an e-beam evaporator for both samples. For the S1, 40 nm of Pd was deposited as the BE, and 40 nm of p^+^-silicon was deposited as the BE for the S2. Then, 60 nm of the IGZO layer was deposited with Ar/O_2_ = 3/2 sccm gas flow using radio frequency (RF) sputtering (150 W). The IGZO layer was deposited with a ratio of In:Ga:Zn = 1:1:1.

**Figure 2:**
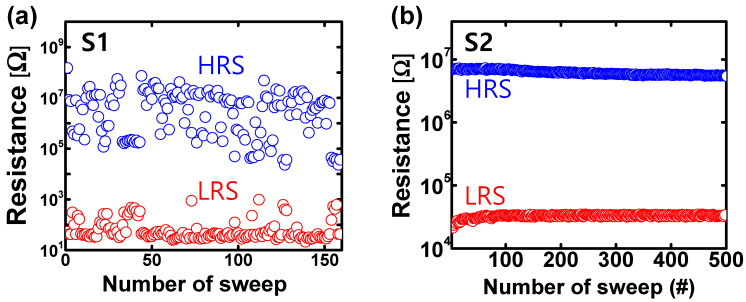
Endurance switching characteristics of (**a**) S1 and (**b**) S2 read at 0.1 V.

For the top electrode (TE), 40 nm of Pd was deposited on both devices using an e-beam evaporator. The electrical characteristics in this work were measured using a semiconductor parameter analyzer (Keithley 4200-SCS). Figure 1c shows the electrical switching characteristics of the S1. The device was formed by a positive voltage sweep of up to 8 V, and 10 mA of compliance current was applied to prevent device overshoot during the forming process. A positive voltage sweep to 6 V was performed for a set operation, whereas a reset operation was performed through a negative voltage sweep to −2.5 V. It is clearly observed that abrupt digital switching occurs during the set and reset processes in the S1 because of the formation and destruction of the CF. In contrast, the forming of the S2 was conducted with a negative voltage sweep to −20 V, as shown in Figure 1d. A positive sweep to 6 V and a negative sweep to −4 V were performed for the set and reset operations, respectively. Unlike the S1, the S2 has gradual switching characteristics, which means the device state can be gradually in the set and reset operations because of the V_O_ generation. Figure 1d,f summarize the cumulative probability of 100 devices for both samples with regard to the high resistive state (HRS) and low resistive state (LRS). It is confirmed that the S1 has a wider state distribution than the S2 because the S1 state changes abruptly by the randomly formed and ruptured CF. These switching behaviors cause a difference in the endurance characteristics of the two samples, as shown in Figure 2. Because the abrupt switching characteristics of the S1 can cause enough stress to the switching layer, the S1 fails to switch after 150 switching cycles between LRS and HRS; however, the S2 can switch over 500 cycles thanks to the gradual switching characteristics.

## 3. Results

**Figure 3:**
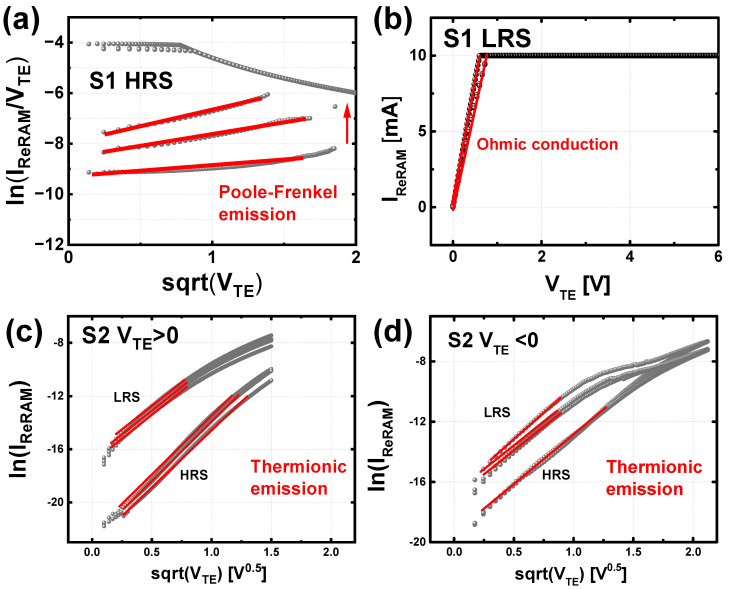
Replotted *I*–*V* switching characteristics of a total of 3 devices. (**a**,**b**) log(*I*/*V*)–sqrt(*V*) plots of S1 and (**c**,**d**) log(*I*)–sqrt(*V*) plots of S2 under positive and negative bias conditions.

The *I*–*V* characteristics of both samples are replotted to investigate the conduction mechanism of each sample, as shown in Figure 3. A total of three devices are verified for the analysis of the conduction mechanism. It is verified that the HRS of the S1 is conducted by the Poole–Frenkel emission since the device current has a relation of $ln\left(\frac{I_{RRAM}}{V}\right)\propto \;\sqrt{V}$, which means that the Schottky barrier (SB) between the IGZO and BE is high enough to suppress thermionic emission from the BE. In addition, the dominant conduction at the LRS of the S1 is confirmed as ohmic conduction, which means that the strong CF is formed during the set operation, and electrons can easily move to the conduction band. In contrast, the dominant conduction mechanism of the S2 is analyzed as thermionic emission regardless of the device state since the *I*–*V* characteristics can be expressed as $ln\left(I_{RRAM}\right)\propto \;\sqrt{V}$ despite the bias polarity. This implies that the device state of the S2 can be controlled by the SB height (*ϕ*_B_) modulation, and the conductance level is modulated in analog grade accordingly.

**Figure 4:**
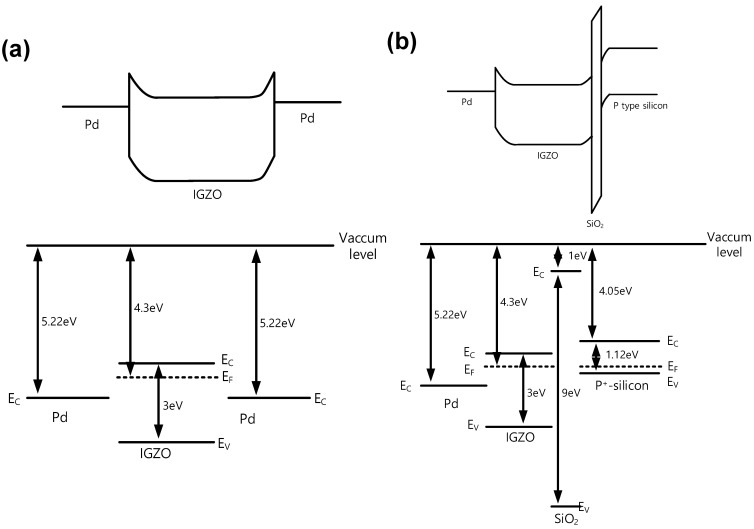
Flat band diagram and energy band diagram in the equilibrium of (**a**) S1 (Pd/IGZO/Pd) and (**b**) S2 (Pd/IGZO/SiO_2_/p-type Si).

To analyze the switching mechanisms of both samples more thoroughly, the flat band diagram and energy band diagram at equilibrium, including work function, electron affinity, and bandgap of the S1 and S2, are illustrated in Figure 4a,b, respectively [47,48]. It is expected that the Schottky barrier near the BE of the S1 is 0.8 eV and can become small similar to the ohmic junction, after the forming process. On the other hand, the SiO_2_ layer inherently formed during the deposition step is placed between the IGZO and BE layers and forms a higher SB than the S1, resulting in a lower-level operation current. It is expected that the device state of the S2 can be controlled by the electron trapping at the interface states between the IGZO and SiO_2_ layers, leading to the gradual switching characteristics.

**Figure 5:**
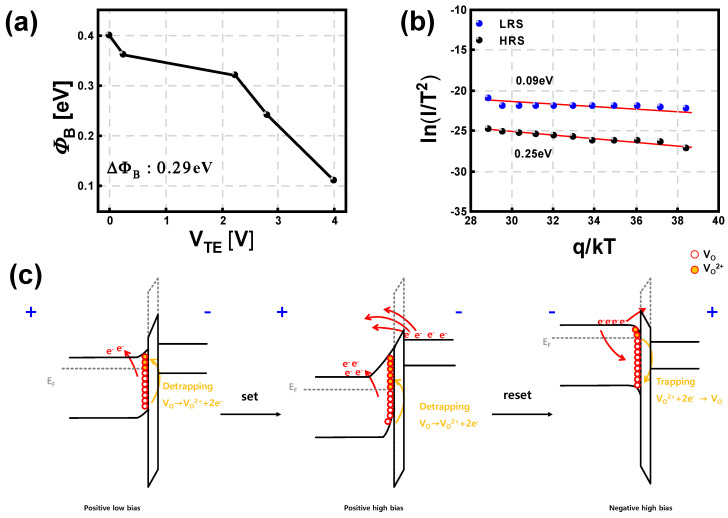
(**a**) *ϕ*_B_ of S2 according to the applied voltage. (**b**) Arrhenius plot of *I*/*T*^2^ for S2 to extract *ϕ*_B_. (**c**) Energy band diagrams of S2 during the set and reset operation.

To confirm the switching mechanism of S2, the *ϕ*_B_ with respect to the TE voltage is extracted, as shown in Figure 5a, using the following equation:

(1) $$ln\left(\frac{I_{RRAM}}{V_{TE}}\right)=\phi_{B}\frac{q}{kT}$$

where *T* is the absolute temperature, *q* is the amount of charge, and *k* is the Planck constant. It is verified that the *ϕ*_B_ is gradually modulated from 0.38 eV to 0.11 eV (0.27 eV of *ϕ*_B_ difference) as the voltage is increased, and accordingly, the device state becomes more conductive by thermionic emission. The *ϕ*_B_ is also extracted from the Arrhenius plot of $ln\left(\frac{I_{RRAM}}{T^{2}}\right)$ for both states (LRS and HRS), as shown in Figure 5b, which also confirms that the *ϕ*_B_ difference is 0.24 eV, and two states can be programmed according to the *ϕ*_B_ modulation. Figure 5c illustrates the change in the *ϕ*_B_ with respect to the applied voltage and movement of electrons using an energy band diagram. The *ϕ*_B_ modulation can be explained by the electric field-induced oxygen ion migration, depending on the bias conditions [49,50]. At a low voltage, electrons cannot move easily because of the SB between the SiO_2_ layer and p-type Si BE. However, as the voltage is increased, oxygen vacancies at the interface of SiO_2_-Si are placed higher than the Fermi level, and they are ionized into V_O_^2+^ and electrons. The electric field generated by V_O_^2+^ ions reduces the *ϕ*_B_, which allows electrons to cross the SB (set process). On the other hand, when a negative voltage is applied, the energy state of the oxygen vacancies is lower than that of the Fermi level, and the ionized V_O_^2+^ becomes neutralized V_O_. Therefore, the lowered *ϕ*_B_ by ionized V_O_^2+^ is restored to its original height, and it becomes difficult for electrons to cross the SB (reset process).

**Figure 6:**
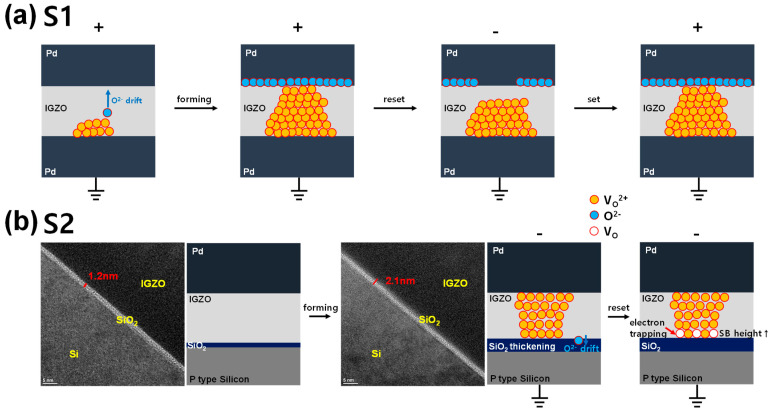
Schematic view of switching operations in (**a**) S1 and (**b**) S2.

The schematic diagram summarizing the conduction mechanism of each device is illustrated in Figure 6. Figure 6a shows the switching operations of the S1. First, when a positive voltage is applied to the TE, oxygen ions drift toward the TE, and a conductive filament composed of V_O_^2+^ is formed. In contrast, when the negative bias is applied to the TE, oxygen ions move back toward the bottom electrode, and the CF is ruptured by the recombination of V_O_^2+^ and oxygen ions, resulting in increased resistance. Once the forming process is conducted, the CF can be easily formed at a lower voltage than the forming process because of the residual filament. Figure 6b shows the switching operations of the S2. As a strong negative voltage is applied to the TE, V_O_^2+^ are generated near the interface between the IGZO and SiO_2_ layers because the oxygen ions drift to the BE. This is because the SiO_2_ layer is more resistant than the IGZO layer, so most of the electrical field is applied across the SiO_2_ layer. Due to the drift of oxygen ions, soft breakdown occurs first in the IGZO layer with low resistance, which leads to the formation of conductive filaments in the IGZO layer [51]. As a result of the oxygen ions drift, the SiO_2_ layer is thickened from 1.2 nm to 2.1 nm after the forming process, as shown in the transmission electron microscopy (TEM) images. The ionized V_O_^2+^ lowers the *ϕ*_B_ and electrons can move easily through thermionic emission, resulting in a decrease in resistance. After the forming process, V_O_^2+^ can become V_O_ by the electron occupancy depending on the bias condition, resulting in the *ϕ*_B_ modulation discussed above.

**Figure 7:**
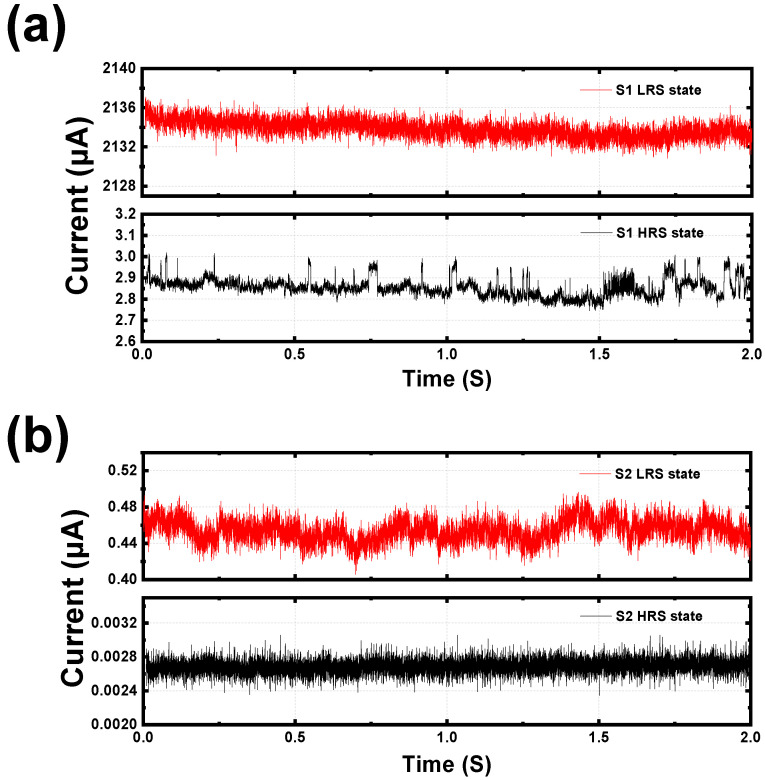
*I*-*t* characteristics of (**a**) S1 and (**b**) S2 under 0.1 V for both HRS and LRS.

Lastly, the RTN signals of both devices were measured to analyze the electron trapping effects depending on the conduction mechanism. RTN is a type of intrinsic random noise, and it refers to a phenomenon in which device current randomly fluctuates by trapping or de-trapping of electrons [52,53]. In general, the RTN signals can be obtained when the CF is ruptured, and the device conduction is dominated by trap-assisted transport, but rarely occurs when the CF is formed since the CF can be hardly affected by electron trapping. The transient characteristics of the device current for the S1 and S2 were measured for 2 s (sample frequency = 10 kHz) under 0.1 V of the TE voltage, as shown in Figure 7a and b, respectively, which confirms that the RTN signals only occur in the HRS of the S1. This implies that the HRS of the S1 is conducted by trap-assisted transport, such as the Pool–Frenkel effect, which is in line with what has been discussed above. In contrast, the noise signals observed in the S2, regardless of the state, are believed to be thermal or 1/f noise rather than RTN signals [54] since its conduction mechanism is thermionic emission for both states, meaning that the S2 is hardly disturbed by intrinsic random noise during the read operation.

## 4. Conclusions

In this study, the conduction mechanisms of abrupt and gradual switching characteristics of IGZO memristors were analyzed. Because of the different BE material, the switching operation of the S1 was obtained by the formation and rupture of the CF, resulting in the abrupt switching, whereas that of the S2 was obtained by the *ϕ*_B_ modulation, resulting in the abrupt switching behaviors. The RTN measurement also confirmed that the S2 was affected by the electron trapping in the HRS because the trap-assisted transport of the S2 was not affected by the electron trapping caused by thermionic emission conduction. We believe that the interface-type switching in the S2 without the trapping effect can be preferred in a neuromorphic system where the memristor device represents floating-point weight values thanks to both gradual switching characteristics and robustness to the intrinsic random noise source.

## Data Availability

The data presented in this study are available on request from the corresponding author.
